# Beyond Neglect: Preliminary Evidence of Retrospective Time Estimation Abnormalities in Non-Neglect Stroke and Transient Ischemic Attack Patients

**DOI:** 10.1038/srep22598

**Published:** 2016-03-04

**Authors:** Essie Low, Sheila G. Crewther, Diana L. Perre, Ben Ong, Robin Laycock, Hans Tu, Tissa Wijeratne

**Affiliations:** 1 Department of Psychology and Counselling, La Trobe University, Bundoora, Victoria, Australia; 2 Neuroscience Research Unit, Sunshine Hospital, Western Health, Victoria, Australia; 3 Department of Psychology, Sunshine Hospital, Western Health, Victoria, Australia; 4 Department of Neurology, Sunshine Hospital, Western Health, Victoria, Australia

## Abstract

Perception of the passage of time is essential for safe planning and navigation of everyday activities. Findings from the literature have demonstrated a gross underestimation of time interval in right-hemisphere damaged neglect patients, but not in non-neglect unilaterally-damaged patients, compared to controls. This study aimed to investigate retrospective estimation of the duration of a target detection task over two occasions, in 30 stroke patients (12 left-side stroke 15 right-side stroke, and 3 right-side stroke with neglect) and 10 transient ischemic attack patients, relative to 31 age-matched controls. Performances on visual short-term and working memory tasks were also examined to investigate the associations between timing abilities with residual cognitive functioning. Initial results revealed evidence of perceptual time underestimation, not just in neglect patients, but also in non-neglect unilaterally-damaged stroke patients and transient ischemic attack patients. Three months later, underestimation of time persisted only in left-side stroke and right-side stroke with neglect patients, who also demonstrated reduced short-term and working memory abilities. Findings from this study suggest a predictive role of residual cognitive impairments in determining the prognosis of perceptual timing abnormalities.

Time perception is referred to as the subjective experience of time, and is often quantified by perception of the duration of elapsed time of a past event^1^. The ability to accurately perceive time is critical for survival, given the need to conceptualise the temporal course of events in preparation and planning for further actions (e,g., to cross a road between oncoming cars, to estimate when to hit/kick a ball in sporting activities)^2^. Thus, if time is subjectively perceived as shorter or longer than its actual passage, this may have significant implications, not just for psychosocial and everyday functioning, but also for safety (e.g., greater risk of being involved in road accidents as a driver or pedestrian).

Neuroimaging studies suggest that timing operations across varying timescales are underpinned by separately distinct neural networks^3^. For example, the circadian clock that operates on a 24-hour timescale is said to be located in the suprachiasmatic nucleus of the hypothalamus, while the operation of sub- or milli-second timing (vital for facilitating automatic timing of actions including motor control) is dependent on cerebellar and motor systems^4^,^5^,^6^. More importantly, less automatic and more cognitively controlled operations of multi-second timing has been suggested to be underpinned by a functionally distributed network encompassing the thalamo-cortico-striatal circuits^3^,^7^. Specifically, this network includes the striatum/basal ganglia, thalamus, supplementary motor area and the prefrontal and parietal cortices^8^,^9^,^10^,^11^,^12^,^13^.

The mechanistic operation behind time perception has traditionally been explained by Treisman’s Information Processing Model^14^ that describes the operation of a pacemaker-accumulator system, or an “internal clock”^7^,^15^,^16^. Generally, this theory posits that internal pulses emitted by a pacemaker mechanism are stored in an accumulator, with the number of stored pulses being proportionally reflective of the perception of time^7^,^10^,^15^. Thus, the greater the number of stored pulses in a given time, the longer the perception of time (which subsequently manifests as time overestimation). More specifically, efficient perceptual timing has also been suggested to be dependent upon several cognitive abilities that facilitate three different stages of the clock model^7^,^17^,^18^,^19^,^20^,^21^. Firstly, storage of pulses in the accumulator has been hypothesized to be dependent on an “attentional switch” that regulates the allocation of attention to the internal timing properties, at the expense of paying attention to the external environment. Thus, more attention paid to time would imply more pulses being transferred to the accumulator. Secondly, the metaphorical description of the accumulator has been equated to a working memory store^7^. Thus, pulses are essentially accumulated in a working memory store for subsequent comparison to a reference, or long-term memory store of previously encoded pulses. This is to facilitate the final decision making stage, where a correct response selection is made following the comparator process. Due to the range of cognitive processes involved, any timing abnormalities could therefore be underpinned by a compromise to either, or a combination of these stages, as a result of limited cognitive resources available to facilitate the clock system^18^,^19^,^22^.

Indeed, impaired perceptual timing has been demonstrated in a variety of clinical populations, including individuals with schizophrenia^19^,^23^,^24^, degenerative diseases such as Alzheimer’s^25^, Parkinson’s^15^, Huntington’s and Multiple System Atrophy^12^, traumatic brain injury^18^, and in neurodevelopmental disorders^26^,^27^. The influence of emotional states on perceptual timing has also been extensively researched, with previous studies highlighting a tendency to overestimate time when individuals are in a depressed, anxious or fearful state, suggesting that such individuals perceive time to pass very slowly^1^,^28^,^29^,^30^,^31^,^32^. Interestingly, a study of individuals with Alzheimer’s disease found both prospective (i.e., participants were prompted in advance to engage in the estimation task) and retrospective (i.e., participants estimated backdated time without prior prompting) time to be significantly underestimated^25^.

To date, there has been relatively little investigation of perceptual timing within the stroke literature, and in particular with individuals following transient ischemic attack (TIA), where transient stroke symptoms resolve within 24 hours^33^,^34^. From the extant literature, findings have consistently demonstrated a *gross underestimation* of time interval in right-hemisphere damaged patients with neglect (RHD-N)^6^,^35^,^36^,^37^ (see Supplementary Table for a summary of studies of perceptual timing after a stroke^6^,^35^,^36^,^37^,^38^,^39^,^40^,^41^). While this underestimation has been frequently interpreted in the context of the pacemaker-accumulator model described above^7^,^15^,^16^, different suggestions have been proposed regarding the specific cause. On one end, the underestimation has been attributed to attentional deficits (reduced allocation of attention to time due to attentional resources being used for the external task at hand) resulting in a deceleration of the pacemaker^35^,^37^. On another end, impairments in spatially maintaining the representation of pulses in the accumulator (i.e., visual working memory deficits), or in comparing this temporary representation of pulses to reference memory, have also been suggested as plausible reasons^6^,^42^.In addition, within non-neglect stroke patients, only one study so far has revealed significantly impaired time estimation (as measured by duration discrimination of auditory tones) following right-hemisphere damage (RHD)^38^, while other studies have only demonstrated a similar trend following RHD^6^,^37^. This disparity in findings may be related to sample sizes, as evidenced by a larger stroke group in Harrington *et al*.^38^ (see Supplementary Table). Currently, there is no evidence yet to suggest impaired timing abilities in stroke patients with left-hemisphere damage (LHD)^41^,^38^. Cerebellar stroke has also not been investigated to a great extent, with Harrington *et al*. noting the lack of support for the role of the cerebellum in regulating timekeeping operations^39^.

Here we aimed to investigate retrospective time estimation of an event (i.e., a target detection n = 0 back task involving serial presentation of visual stimuli) in the multi-second timescale, in neglect and non-neglect stroke patients, and in TIA patients. Estimation was examined retrospectively, as such a situation is generalisable to the perception of the passage of time in naturalistic settings, where individuals often do not consciously allocate additional attentional resources to the timing properties of an event^22^,^25^. Given that a *retrospective* estimation of time data is unique to a particular setting and cannot be easily replicated across successive trials, we collected a second estimate of time spent on the same task at a follow-up three monthly clinical visit. In view of suggestions that timing abilities may be related to the interplay between paying attention to time and to the external environmental/stimulus properties, as well as to working memory^7^,^16^,^18^,^19^,^20^,^21^, we also explored participants’ sustained attention and reaction time performance on the same target detection task (as indicators of adequacy of externally driven attentional allocation to the task), in addition to performances on visually-based short-term and working memory tasks. We hypothesised that time estimation in RHD-N and RHD patients would be significantly reduced compared to controls, while time estimation in LHD patients would not be significantly different to controls. We also hypothesised that TIA patients would not perform any differently to controls, given less severe symptomatology. To our knowledge, this is the first study to investigate perceptual timing abilities in TIA patients.

## Methods

### Participants

This study is part of a larger project that was approved by the Western Health Low Risk Human Research Ethics Panel (HREC/13/WH/105) and the La Trobe University Human Research Ethics Committee. All participants provided written informed consent prior to their participation, in accordance with the Declaration of Helsinki methods were carried out in accordance with the approved guidelines.

Thirty ischemic stroke patients, comprising 12 LHD patients (*M age* = 54.50, *SD* = 9.02), 15 RHD patients (*M age* = 58.87, *SD* = 9.01) and 3 RHD-N patients (*M age* = 66.67, *SD* = 9.50), and another 10 TIA patients (*M age* = 59.70, *SD* = 9.41) participated in this study. Duration between hospital admission and time of first participation at the outpatient clinic was one to 16 months for stroke patients (with the exception of one LHD patient who was first seen 27 months post-stroke), and one to eight months for TIA patients. Inclusion criteria required that patients: 1) were aged between 40 and 80 years; 2) had a cerebral infarct; 3) had adequate ability to understand English, and therefore, the capacity to provide consent and understand task instructions; 4) had not been diagnosed with any neurological, psychiatric or neurodevelopmental condition prior to the stroke; and 5) were not, at the time of recruitment, diagnosed with a psychiatric or degenerative condition. Diagnosis of stroke or TIA was made by the neurologist based on accepted clinical characteristics and symptoms, and where possible, neuroimaging data^43^. Neglect in the three patients (i.e., RHD-N) was identified using neuropsychological tasks of line cancellation^44^, shape cancellation^45^, and clock drawing^46^, and in some patients, corroborated by overt signs on presentation or on a double simultaneous stimulation examination. Based on qualitative observations, all patients demonstrated adequate expressive and receptive language to understand verbal information, respond to instructions, and to consent to participate in testing. Patients were seen again approximately three months following the initial session (*M duration* = 108 days, *SD* = 29.97), during their clinical visit. Due to attrition, sample sizes reduced to n = 9 LHD, n = 10 RHD, n = 9 TIA and n = 3 RHD-N patients.

Thirty one neurologically healthy controls (*M age* = 55.07, *SD* = 8.69) were recruited by advertisement of flyers at the hospital and community centres, and by word of mouth to patients’ relatives and next of kin. Inclusion criteria were similar to that of the patient group, apart from criteria relating to neurological events. Controls were matched to patients by age and gender. Excluding RHD-N patients (due to small sample size), a one-way ANOVA revealed no significant difference in age between groups, *F*(3, 64) = 1.25, *p* = 0.30, *η*^2^ = 0.06. Chi-square test revealed no significant difference in gender proportions in the stroke and TIA samples, compared to the control group (43% male), *X*^2^ (1, *n* = 37)=1.85, *p* = 0.17. One-way ANOVA revealed a significant difference in mean number of years of education between groups, *F*(3, 64) = 3.60, *p* = 0.02, *η*^2^ = 0.14, with the LHD group having a lower mean number of years of education (*M *= 10.50, *SD *= 2.61) compared to the control group (*M *= 13.52, *SD *= 2.16). Based on completion of the Edinburgh Handedness Inventory (EHI)^47^, two LHD patients were confirmed as left-handed, while one LHD and one RHD patient were ambidextrous. The left-handed patients were matched to two left-handed controls. Further demographic and clinical information is presented in Table 1.

Patients and controls were screened for non-verbal intelligences (and as such, any possible pre-morbid low intellectual level) using the Raven’s Coloured Progressive Matrices (RCPM)^48^. The RCPM was also used to identify any significant visually-based cognitive deficits, including visuo-spatial integration and reasoning. Maximum score is 36, with a higher score indicating better performance. Participants were excluded if they obtained a score of lower than 20/36. The 21-Item Depression Anxiety and Stress Scale (DASS-21) was included to assess levels of depressive, anxiety, and stress symptomatology^49^. Maximum score for each scale is 21, with a higher score indicating greater self-report of symptoms. Participants who endorsed ‘severe’ or ‘extremely severe’ levels of depressive symptoms, as reflected by a score of 11/21 and above, were excluded. On this background, there were two additional stroke participants (one LHD and one RHD) recruited, whom we excluded retrospectively from this study. Note that one RHD-N patient, two LHD patients and three controls could not complete the DASS-21 as they were of non-English speaking background; however, subjective questioning indicated no psychiatric history nor a current experience of low mood or anxiety. Excluding RHD-N patients (due to small sample size), a one-way ANOVA revealed no significant difference between groups in mean scores for depressive symptoms, *F*(3, 59)* *= 2.40, p = 0.08, *η*^2^* *= 0.11, and stress symptoms, *F*(3, 59)* *= 1.71, p = 0.18, *η*^2^* *= 0.08. There was a significant difference in mean scores for anxiety symptoms, *F*(3, 59)* *= 3.94, p = 0.01, *η*^2^* *= 0.17, with the TIA group reporting higher scores (*M *= 3.60, *SD *= 2.88) compared to controls (*M *= 1.18, *SD *= 1.28). The Purdue Pegboard Task was administered to examine manual dexterity of the upper limbs. Participants were requested to insert as many pegs into the pegboard holes in 30 seconds, using their dominant and non-dominant hands, respectively. Higher scores (i.e., total number of pegs) reflect greater dexterity. As this task was administered to patients only on the second occasion, performances were examined from a smaller sample group. Manual dexterity scores are presented in Table 1.

### Materials and Procedure

#### Target Detection (n = 0 back) Task and Time Estimation

The target detection task was developed using VPixx software (vpixx.com) and displayed on a Macbook Pro with a 13-inch monitor. This task consisted of a series of 34 cartoon faces that vary in shape (i.e., 9 star-shaped faces, 9 round-shaped faces, 8 square-shaped faces, and 8 oval-shaped faces), presented centrally with each face subtending approximately 10 by 10 degrees of visual angle at 57 cm from the monitor. Prior to commencing, participants were seated comfortably at approximately 57 cm away from the laptop screen and given instructions about the procedure. The face stimuli were presented sequentially for 1.5 seconds per stimulus, with a 1 second delay between stimuli. Participants were requested to hit the spacebar as quickly as possible, using their dominant hand, each time they saw a star-shaped face (target face) appear on the screen, an event that occurred on 26.5% of trials. In total there were 34 trials (corresponding to the 34 faces) with 9 targets appearing in a random sequence. Following completion of the 85-second task, participants were asked to guess “how long do you think the task went on for?” in seconds.

#### Visual Digit Span Forward (VSF) and Backward (VSB)

The VSF and VSB were adapted from the Auditory Digit Span subtest of the Wechsler Adult Intelligence Scale 4^th^ Edition^50^. The forward span is known to measure attention in the context of immediate recall of information from short-term memory, while the backward span places demands on both attention and working memory abilities^51^. During the VSF, participants were shown a string of 6 × 6 cm cue cards, depicting a large-font number on each card. Cards were presented at a rate of one every two seconds, with each card being exposed for one second, followed by a time lapse of one second before the next card was presented. Following presentation of the string of cards in random order, participants were instructed to recall the numbers in the same order that was shown. Administration rules for the task were similar to that of the WAIS-IV Auditory Digit Span, with the string of cards increasing in length across trials, from a two-card string to a maximum of eight cards. The task was terminated following two consecutive incorrect responses on the same number string. Similarly, the same administration rules were adhered to for the VSB, although on this occasion, participants were instructed instead to recall the numbers in reverse order.

The above materials were administered to both control and patient participants on the first occasion. For patients, target detection and time estimation were administered again on the second occasion. Due to attrition, performances on the second occasion were examined from a smaller sample size (see Methods: Participants). In addition, due to ethical obligations and adherence to the approved protocol, VSF and VSB data were not collected for participants who presented with slow speed of processing on the first session, due to time constraints. Performance on these tasks was therefore examined from a smaller sample size of n = 10 LHD, n = 12 RHD, n = 9 TIA, n = 3 RHD-N and n = 29 controls.

### Operationalisation of Variables

#### Mean Time Estimate (Mean TE)

Initial and three-month Mean TE was calculated by averaging the time estimate (in seconds) across participants within each group.

#### Sustained Attention

Ability to sustain attention was measured by accuracy in identifying the targets, as operationalised by the *number of hits* for star-shaped faces. Full score is a total of nine hits, confirming that participants were generally able to sustain their attention throughout the task. All patients scored nine hits, apart from 2 RHD and 1 TIA patient who scored eight. All control subjects also scored nine hits. Given the narrow range of scores (i.e., 0–9) and the fact that participants performed at ceiling, hit scores were not subjected to subsequent statistical analyses.

#### Mean Motor Reaction Time (Mean MRT)

Mean MRT was calculated first, by averaging the reaction time for responding to the correct target faces (i.e., by hitting the spacebar) for each participant, then followed by averaging the reaction time across participants within each group.

#### Mean VSF

Scoring rules were adapted from the WAIS-IV Auditory Digit Span^50^. Total score was the sum of correct responses across trials. Each participant’s score was then averaged to provide a Mean VSF for each group.

#### Mean VSB

Scoring rules were adapted from the WAIS-IV Auditory Digit Span^50^. Total score was the sum of correct responses across trials. Each participant’s score was then averaged to provide a Mean VSB for each group.

### Statistical analysis

Given our a-priori hypotheses, one-way ANOVA with planned contrast comparison was performed for each Mean TE measure, to determine any significant difference and the strength of difference between clinical groups (LHD, RHD, RHD-N, TIA) and the control group. This was repeated for Mean MRT, Mean VSF and Mean VSB.

Pearson’s correlational analysis was conducted between the initial Mean TE with other performance variables, including Mean MRT, Mean VSF and Mean VSB, to investigate the associations between timing abilities with these variables. For Mean VSF and Mean VSB data, correlational analysis was performed for all participants combined (to increase statistical power), given the relatively smaller sample sizes.

All analyses were performed using IBM SPSS Statistics 22.

## Results

Distribution of Mean TE, Mean MRT, Mean VSF and Mean VSB scores for LHD, RHD, TIA and control groups, including skewness and kurtosis, were within appropriate ranges. This was not examined for the RHD-N group (n = 3).

### Initial Mean Time Estimation

**Figure 1:**
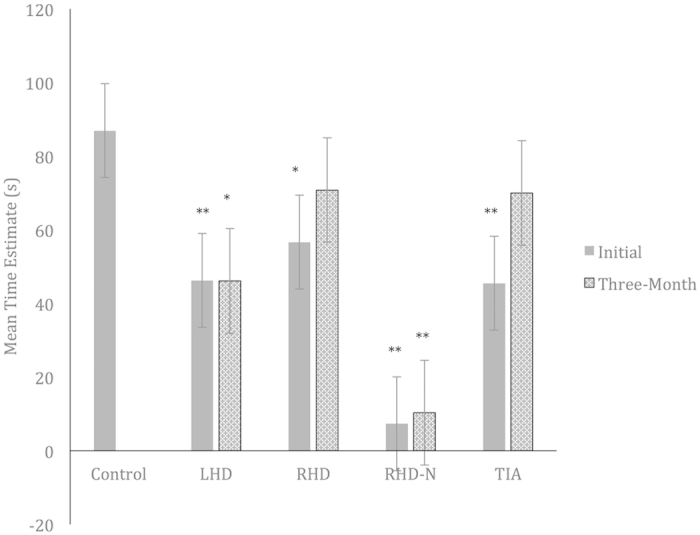
Mean time estimate for clinical and control groups. LHD = Left-Hemisphere Damage Stroke Patients; RHD = Right-Hemisphere Damage Stroke Patients; RHD-N = Right-Hemisphere Damage Stroke Patients with Neglect; TIA = Transient Ischemic Attack Patients. Asterisks indicate where performance is significantly different to that of the control group, *p < 0.05, **p < 0.01.

One-way ANOVA (using the Welch test given that the assumption of homogeneity of variance was not met) revealed a significant difference in the initial Mean TE for groups, *F*(4, 27)* *= 38.43, *p *< 0.001. Further planned contrast comparisons revealed that task duration was significantly underestimated in LHD (*M *= 46.25, *SD *= 17.98, *p *= 0.005, *Cohen’s d *= −0.98), RHD (*M *= 56.67, *SD *= 24.62, *p *= 0.023, *d *= −0.73), RHD-N (*M *= 7.33, *SD *= 3.06, *p *= 0.002, *d *= −1.93) and TIA (*M *= 45.50, *SD *= 21.66, *p *= 0.008, *d *= −1.00) groups, compared to controls (*M *= 86.94, *SD *= 56.71) (Fig. 1).

### Three-Month Mean Time Estimation

One-way ANOVA (using the Welch test) revealed a significant difference in the three-month Mean TE score for groups, *F*(4, 20)* *= 23.39, *p *< 0.001. Further planned contrast comparisons revealed that task duration was significantly underestimated in LHD (*M *= 46.11, *SD *= 33.61, *p *= 0.04, *d *= −0.81) and RHD-N (*M *= 10.33, *SD *= 0.58, *p *= 0.01, *d *= −1.53) groups, compared to controls. This significant underestimation was not observed in RHD (*M *= 70.80, *SD *= 37.31, *p *= 0.38, *d *= −0.32) and TIA (*M *= 70.00, *SD *= 56.51, *p *= 0.38, *d *= −0.34) groups (Fig. 1).

### Mean Motor Reaction Time

**Figure 2:**
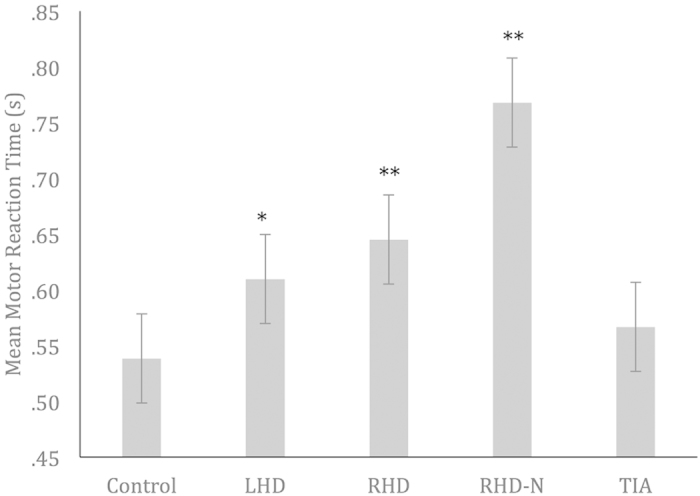
Mean motor reaction time for clinical and control groups. LHD = Left-Hemisphere Damage Stroke Patients; RHD = Right-Hemisphere Damage Stroke Patients; RHD-N = Right-Hemisphere Damage Stroke Patients with Neglect; TIA = Transient Ischemic Attack Patients. Asterisks indicate where performance is significantly different to that of the control group, *p < 0.05, **p < 0.01.

One-way ANOVA (using the Welch test) revealed a significant difference in Mean MRT for groups, *F*(4, 11)* *= 4.14, *p *= 0.027. Further planned contrast comparisons revealed that motor reaction time was significantly greater in LHD (*M *= 0.61, *SD *= 0.06, *p *= 0.04 , *d *= 0.75), RHD (*M *= 0.65, *SD *= 0.12, *p *= 0.000, *d *= 1.13), and RHD-N (*M *= 0.77, *SD *= 0.19, *p *= 0.000, *d *= 2.43) groups, compared to controls (*M *= 0.54, *SD *= 0.07), suggesting that LHD, RHD, & RHD-N patients required a significantly longer time to identify and respond to the target faces. This difference was not observed in the TIA group (*M *= 0.57, *SD *= 0.11, *p *= 0.42, *d *= 0.30) (Fig. 2).

### Mean Visual Short Term Memory

**Figure 3:**
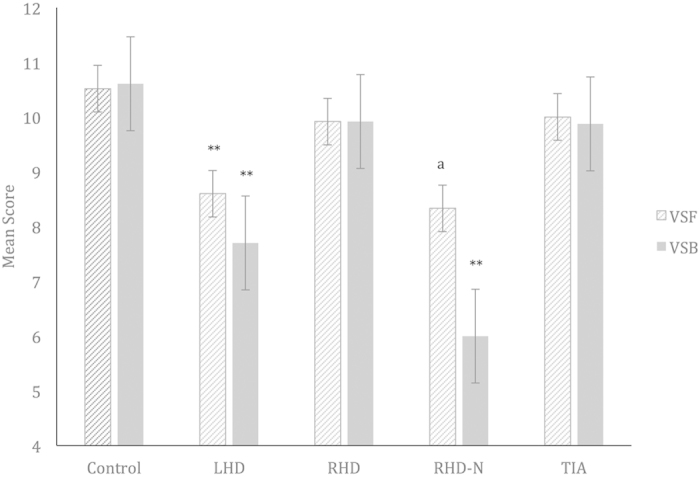
Mean visual digit span forward and backward scores for clinical and control groups. VSF = Visual Digit Span Forward; VSB = Visual Digit Span Backward; LHD = Left-Hemisphere Damage Stroke Patients; RHD = Right-Hemisphere Damage Stroke Patients; RHD-N = Right-Hemisphere Damage Stroke Patients with Neglect; TIA = Transient Ischemic Attack Patients. Asterisks indicate where performance is significantly different to that of the control group, *p < 0.05, **p < 0.01. ^a^large effect size.

One-way ANOVA revealed no significant difference in Mean VSF scores for groups, *F*(4, 58)* *= 1.59, *p *= 0.19. Note however, that planned contrast comparisons revealed significantly lower Mean VSF scores in the LHD group (*M *= 8.60, *SD *= 1.90, *p *= 0.03, *d *= −0.81), compared to controls *(M *= 10.52, *SD *= 2.25). This significantly poorer performance was not observed in RHD (*M *= 9.92, *SD *= 2.84, *p *= 0.46, *d *= −0.25), RHD-N (*M *= 8.33, *SD *= 0.58, *p *= 0.13, *d *= −0.92) and TIA (*M *= 10.00, *SD *= 2.74, *p *= 0.57, *d *= −0.22) groups. However, the large effect size for the RHD-N group suggests that an effect would be observable with a larger sample size (Fig. 3).

### Mean Visual Working Memory

One-way ANOVA revealed a significant difference in Mean VSB scores for groups, *F*(4, 56)* *= 3.96, *p *= 0.007. Further planned contrast comparisons revealed significantly lower Mean VSB scores in the LHD (*M *= 7.70, *SD *= 1.57, *p *= 0.003, *d *= −1.13) and RHD-N (*M *= 6.00, *SD *= 2.00, *p *= 0.005, *d *= −1.79) groups, compared to controls *(M *= 10.61, *SD *= 2.77). This significantly poorer performance was not observed in RHD (*M *= 9.92, *SD *= 2.75, *p *= 0.44, *d *= −0.27) and TIA (*M *= 9.88, *SD *= 2.70, *p *= 0.48, *d *= −0.28) groups (Fig. 3).

### Associations between Time Estimation and Other Task Variables

Correlational analysis revealed no significant associations between the initial Mean TE with Mean MRT for LHD (r* *= 0.39, p* *= 0.24), RHD (r* *= −0.04, p* *= 0.89), RHD-N (r* *= 0.59, p* *= 0.60), TIA (r* *= −0.28, p* *= 0.44) and control (r* *= 0.06, p* *= 0.76) groups. For all participants combined, there was also no significant association between the initial Mean TE with forward span (*r *= 0.17, *p *= 0.20, *n *= 63), although a trend towards significance was observed between the initial Mean TE with backward span (*r *= 0.24, *p *= 0.06, *n *= 61). This suggests that an increase in time estimates may be associated with better working memory.

## Discussion

The aim of the current study was to investigate retrospective time interval estimation of an event following both neglect and non-neglect ischemic stroke, and TIA, at two time points, three months apart. The initial time estimation results revealed that both RHD-N and RHD groups significantly underestimated time compared to controls, thus providing support for the first hypothesis. Notably, neglect patients appeared to underestimate time by a very large margin, with their subjective perception of the time that elapsed being an average of *seven* seconds only, compared to the actual duration of 85 seconds. However, this finding was not unanticipated and confirmed previous studies^6^,^35^,^36^,^37^.

Similarly, the significant time underestimation exhibited by non-neglect RHD patients, albeit less severe, was also supported by previous studies revealing the same pattern of performance^6^,^37^. In particular, Danckert *et al*.^6^ investigated verbally reported estimation of RHD patients at variable time intervals, and found patients to significantly underestimate time at the longer interval of 60 seconds (while only a trend was demonstrated for shorter intervals), which compares well with the current findings. It was further suggested in another study^19^, that such pattern of results are likely modulated by the cognitive demands associated with time interval processing, whereby the discrimination of shorter time intervals was argued to require less cognitive resources (such as working memory) compared to longer time intervals. These findings therefore imply that impairments in timing behaviour may increase in an exponential manner as a function of longer time intervals. Interestingly, although Morin *et al*.^41^ (see Supplementary Table) did not find significant timing impairments in a large sample of RHD patients despite examining time interval within the longer, *minute* timescale, examination of an interval within a different timescale range altogether, may be tapping into different neural regions^3^.

Our first collection of time estimation data also revealed significant underestimations made by LHD and TIA patients (this was again less severe than for neglect patients), and thus contradicted the second and third hypotheses. By comparison to RHD patients, there were only two studies^38^,^41^ that have investigated timing behaviour in LHD patients, and neither found any deviant performance. Again, both studies employed different timescales of within the minute and millisecond range, respectively, which is likely to contribute to the differences in findings. Importantly, the underestimation by TIA patients was largely not within our expectations, given that our clinical definition of TIA indicates less than 24 hours of behavioural/physical symptoms and no pathological lesions on imaging. To our knowledge, this is the first study to demonstrate time estimation deficits in TIA patients, and given the absence of focal lesions, future research is essential to explore other plausible factors, including white matter disease and neurochemical changes.

With regards to time estimation performance on the second occasion, the RHD-N group again demonstrated an excessive underestimation of time, as evidenced by a mean estimate of 10 seconds and with minimal variation (*SD * = ±0.58 s). While the reliability of a single measure collected at a single time point may be called into question, corroborative results from the second occasion strongly indicates a genuine presence of perceptual timing impairments, at least in this patient group. Furthermore, persistent underestimation was also demonstrated by LHD patients, although a larger variance (SD = ±0.34 s) was observed on the second occasion. The larger variance likely indicates that some patients, at least, were making more accurate estimates, which may be a learning effect or a sign of recovery post-vascular event. On the contrary, the initial underestimation made by both RHD and TIA groups appeared to have attenuated on the second occasion (Fig. 1). This notable change in performance certainly suggests the potential for recovery of timing abilities, but the fact that only single measures could be obtained per session, necessitates that such conclusions require further validation.

We reported two common outcome measures of the target detection task that may concurrently explain timing behaviour. Firstly, all patients demonstrated optimal ability to sustain their attention for the brief period of the task, as evidenced by their perfect, or almost perfect hit scores. This suggests that they were fully and actively engaging their visual top-down driven attention to the task. If interpreted in terms of the pacemaker-accumulator model^16^,^21^, full engagement on the task would imply more limited attentional resources available to internally monitor time passage, resulting in a deceleration of the pacemaker. In fact, it has been shown in the lesioned brain that more than normal levels of conscious energy are required to concentrate and stay on task, often manifesting as an increase in neural activity during functional imaging studies^52^,^53^. Our second measure was motor reaction time, with analyses indicating that all stroke (LHD, RHD, and RHD-N) groups required a significantly longer time to respond to the targets compared to controls. Based on this finding, we speculate that reaction time may not be an optimal measure of externally driven attention to the task, since this measure is heavily dependent on psychomotor response. Furthermore, our speculation is given credibility by the clinically recorded Pegboard scores, which indicated marginally reduced upper limb dexterity in our stroke patients (Table 1).

A visually presented digit span task was administered to better profile the role of cognitive functions on timing behaviour. Findings revealed poorer performances on both forward and backward span, by the LHD and RHD-N groups, but not by the RHD and TIA groups compared to controls. This is an interesting finding, particularly given that the LHD and RHD-N patients with reduced short-term and working memory abilities also demonstrated persistent time underestimation over the three-month interval (Figs 1 and 3). By comparison, the RHD and TIA patients, who exhibited less pronounced short-term and working memory deficits, demonstrated a degree of change towards more accurate estimation on the second occasion. Several explanations could be considered in light of these results. Firstly, the consistency in timing and cognitive performance suggest that timing impairments are intrinsically linked to the underlying attentional and working memory deficits, which is in line with the pacemaker-accumulator model. More importantly, the current results indicate a likely predictive role of the *extent of cognitive deficits* in determining prognosis of timing abnormalities, such that long-standing timing impairments may more likely occur with greater cognitive compromise, and vice-versa. Given that both simple attentional and working memory abilities were concurrently reduced in LHD and RHD-N patients (as evidenced by poor performance on both forward and backward span), this finding also suggests that timing abnormalities are more likely due to a combination of cognitive processing deficits affecting a range of pacemaker-accumulator processes, rather than to a single deficiency of the pacemaker-accumulator system.

Correlational analyses were performed between time estimation with other task variables, and revealed that an increase in time estimates was likely associated with better working memory. Although only marginally significant, the direction of the relationship still highlights a vital role of working memory in facilitating efficient perceptual timing – this is furthermore consistent with previous studies that have demonstrated the same associations in TBI and schizophrenia samples^18^,^19^,^54^. While there is also evidence to suggest that visuo-spatial working memory underpins timing behaviour (i.e., that time pulses are represented spatially) more so than verbal working memory^6^, the generalizability of the current results may be limited by the approach to task administration that was employed, which also engages language centres in addition to visual regions.

Despite some new insights on perceptual timing following stroke and TIA, several limitations should be acknowledged. Firstly, TIA patients were assessed at an earlier time following the transient episode (118 days) compared to stroke patients (202–231 days). However, given that time since TIA did not correlate with time estimates, we did not anticipate that this would have a substantial impact on performance. Secondly, TIA patients appeared to report significantly higher levels of anxiety symptoms on the DASS-21 compared to controls (and although no significant difference was found between groups in scores for depressive symptoms, the effect size was relatively large). An Analysis of Covariance was performed to address this matter, but did not appear to alter the current time estimate results even when scores on the depression and anxiety symptom scales were controlled/co-varied. Finally, the likelihood of some patients remembering the estimation task from the first occasion is possible, and may impact the validity of the second round data as being retrospective. However, obtaining a reliable retrospective measure is evidently a challenge, given that any repeat trial will inadvertently introduce an anticipatory effect and a bias towards attention to time.

In summary, the current study provides preliminary evidence of perceptual time underestimation, not just in neglect patients, but also in unilaterally lesioned non-neglect stroke and TIA patients. Importantly, the findings of a timing deficit in TIA patients, as well as a likely interaction between cognition with prognosis of timing deficits are of particular significance, and will benefit from future research. Given considerable evidence for timing mechanisms to be related to cognitive efficiency^5^,^18^,^22^,^23^, future research should also manipulate cognitive demands (e.g., by incorporating low and high attention-demanding tasks) to further determine how competition for resources may impact on timing behaviour. Finally, given the functional impacts of abnormal perceptual timing^2^, we argue that characterisation of time perception may prove useful in informing the efficacy of rehabilitative approaches to assist with return of functional independence, and to improve quality of life.

## Additional Information

**How to cite this article**: Low, E. *et al*. Beyond Neglect: Preliminary Evidence of Retrospective Time Estimation Abnormalities in Non-Neglect Stroke and Transient Ischemic Attack Patients. *Sci. Rep.* **6**, 22598; doi: 10.1038/srep22598 (2016).

## Supplementary Material

Supplementary Information

## Figures and Tables

**Table 1:** Group demographics and clinical information.

	Control	LHD	RHD	RHD-N	TIA
Age (years)	55.07 (8.69)	54.50 (9.02)	58.87 (9.01)	66.67 (9.50)	59.70 (9.41)
Education (total years)	13.52 (2.16)	10.50 (2.61)	12.20 (3.05)	7.00 (3.00)	12.50 (3.89)
Gender (% male)	43	50	47	100	70
DASS-21 Depression	1.39 (2.06)	3.67 (3.50)	2.54 (2.50)	0.00 (.00)	3.20 (3.65)
DASS-21 Anxiety	1.18 (1.28)	2.42 (2.71)	2.31 (1.80)	0.50 (.71)	3.60 (2.88)
DASS-21 Stress	3.11 (2.81)	4.42 (3.15)	4.62 (2.69)	0.50 (.71)	5.50 (4.55)
RCPM	33.32 (2.01)	28.25 (3.74)	29.13 (4.16)	22.00 (3.00)	30.10 (3.67)
Pegboard Index^b^	13.03 (1.42)	11.68 (1.85)	11.06 (1.59)	10.75 (3.18)	11.17 (1.89)
Time post-stroke/TIA (days)	– (−)	202.36^a^ (106.38)	230.93 (138.78)	179.00 (63.91)	118.30 (56.97)
Severity^c^ (FIM pre-rehab)	– (−)	89.25 (−)	71.67 (−)	77.00 (−)	– (−)
Severity^c^ (FIM post-rehab)	– (−)	120.25 (−)	104.33 (−)	111.00 (−)	– (−)

Note DASS-21 = 21-Item Depression Anxiety Stress Scale; RCPM = Raven’s Coloured Progressive Matrices; FIM = Functional Independence Measure; LHD = Left-Hemisphere Damage Stroke Patients; RHD = Right-Hemisphere Damage Stroke Patients; RHD-N = Right-Hemisphere Damage Stroke Patients with Neglect; TIA = Transient Ischemic Attack Patients. Dashes indicate where descriptive statistics were not applicable.

^a^Time post-stroke Mean and SD data were for n = 11 patients. The remaining patient was assessed at 817 days post-stroke. This patient was included into the study as the data did not largely alter the results.

^b^Pegboard Index was calculated by averaging dominant and non-dominant hand performance (total pegs placed in 30 seconds for each hand respectively).

^c^As FIM assessment is not undertaken routinely at Western Health, scores were only available for stroke patients who had been admitted to the subacute rehabilitation ward following acute admission. Reported scores were from a sample size of n = 4 LHD, n = 3 RHD and n = 2 RHD-N patients.
