# Supplementary material for: Beyond Neglect: Preliminary Evidence of Retrospective Time Estimation Abnormalities in Non-Neglect Stroke and Transient Ischemic Attack Patients
Source: Sci Rep. 2016 Mar 4;6:22598. doi: 10.1038/srep22598 (PMC4778116; doi:10.1038/srep22598)
Supplement: Supplementary Information [file srep22598-s1.pdf]

Beyond Neglect: Preliminary Evidence of Retrospective Time Estimation Abnormalities in Non-Neglect Stroke and Transient Ischemic Attack Patients

Essie Low, Sheila G. Crewther, Diana L. Perre, Ben Ong, Robin Laycock , Hans Tu & Tissa Wijeratne

Supplementary Table. Experimental details of studies investigating perceptual timing following stroke.

Studies	Primary aim/question of interest	Clinical group and sample size	Operationalisation of the dependent measure	Paradigm	Modality	Timescale	Results
Basso et al. (1996)	To investigate if memory-based models or internal clock models account for time distortion	RHD-N (case study)	Estimation of the duration of stimuli presentation in the neglected and non-neglected space	Prospective	Visual	Millisecond	Stimuli duration was overestimated in neglected space but underestimated in non-neglected space
Harrington et al. (1998)	To investigate the role of the cerebral cortex on timekeeping operations	RHD (n=18)<br><br>LHD (n=19)	Time estimation – discriminate time lengths of two tone pairs<br><br>Frequency estimation – discriminate pitch of two tone pairs	Prospective	Auditory	Millisecond	When frequency perception deficits were controlled, only RHD individuals showed time perception deficits
Harrington et al. (2004)	Role of cerebellum on	Cerebellar (n=21)	Time reproduction – reproduce 300ms and	Prospective	Auditory	Millisecond	Increased variability in time reproduction but no

	timing abilities	Controls (n=30)	600ms time interval via tapping<br><br>Time estimation – discriminate time lengths of two tone pairs				significant deviance in time estimation
Morin et al. (2005)	To investigate relationship between right brain injury with clock time estimation	RHD (n=21)<br><br>LHD (n=27)<br><br>TIA controls (n=20)	Estimation of clock-time ("what time is it in your opinion?")<br><br>Estimation of the duration of a short interview (~26 minutes)	Retrospecti ve	Non-specific	Minute	RHD significantly overestimate clock-time compared to TIA controls, but not LHD<br><br>No significant deviance in estimation of interview duration for RHD and LHD groups compared to TIA controls
Danckert et al. (2007)	To investigate the perception of the passage of time in neglect	RHD-N (n=8)<br><br>RHD (n=6)<br><br>Controls (n=8)	Estimation of visual illusory motion stimulus on laptop screen	Prospective	Visual	Second	RHD-N significantly underestimated stimulus duration<br><br>RHD showed a trend in underestimating stimulus duration, but performance was better than RHD-N
Merrifield et al. (2010)	Role of sensory modalities on timing abilities	RHD-N & LHD (case	Estimation of the duration of white noise and illusory	Prospective	Visual and auditory	Second	RHD-N patient underestimated task duration

		study)	motion stimulus				No deviance in duration estimation for LHD patient
Calabria et al. (2011)	To investigate if time, space, and number share the same neural basis – the right parietal cortex	RHD-N (n=6)<br><br>RHD (n=8)<br><br>Controls (n=8)	Discriminating between the lengths of two time intervals	Prospective	Auditory	Millisecond	RHD-N performed significantly worse than RHD and controls
de Montalembert & Mamassian (2012)	To investigate simultaneous (rather than sequential) processing of time perception	RHD-N (n=8)<br><br>Controls (n=20)	Discrimination between the duration of two stimuli when presented simultaneously and sequentially	Prospective	Visual	Millisecond	Performance (for controls) was significantly worse for simultaneous presentation.<br>This was the same for RHD-N, although patients were also more impaired when stimuli was presented first on the right visual field, followed by left

Studies are listed in accordance to year of publication. Studies have been cited in the main article, with full details obtainable from reference list of the article. RHD-N = right hemisphere-damaged patients with neglect; RHD = right hemisphere-damaged patients without neglect; LHD = left hemisphere-damaged patients without neglect.

Note: List of studies was obtained in a systematic approach to identify relevant articles that investigated timekeeping operations following a stroke and a TIA. The following search terms were used on Pubmed and PsycInfo databases: 1) “stroke” AND “time estimat*/perception”; 2) “stroke” AND “temporal estimat*/perception”; and 3) ‘stroke” AND “duration estimate*/perception”. “Stroke” was then replaced with ““transient” and “neglect” to incorporate studies that may have investigated timing abilities in individuals following a TIA and in stroke patients with neglect.
